# A Coastal Cline in Sodium Accumulation in *Arabidopsis thaliana* Is Driven by Natural Variation of the Sodium Transporter AtHKT1;1

**DOI:** 10.1371/journal.pgen.1001193

**Published:** 2010-11-11

**Authors:** Ivan Baxter, Jessica N. Brazelton, Danni Yu, Yu S. Huang, Brett Lahner, Elena Yakubova, Yan Li, Joy Bergelson, Justin O. Borevitz, Magnus Nordborg, Olga Vitek, David E. Salt

**Affiliations:** 1 United States Department of Agriculture–Agricultural Research Service, Plant Genetics Research Unit, Donald Danforth Plant Sciences Center, St. Louis, Missouri, United States of America; 2 Department of Horticulture and Landscape Architecture, Purdue University, West Lafayette, Indiana, United States of America; 3 Department of Statistics, Purdue University, West Lafayette, Indiana, United States of America; 4 Molecular and Computational Biology, University of Southern California, Los Angeles, California, United States of America; 5 Department of Ecology and Evolution, University of Chicago, Chicago, Illinois, United States of America; 6 Gregor Mendel Institute of Molecular Plant Biology, Austrian Academy of Sciences, Vienna, Austria; The University of North Carolina at Chapel Hill, United States of America

## Abstract

The genetic model plant *Arabidopsis thaliana*, like many plant species, experiences a range of edaphic conditions across its natural habitat. Such heterogeneity may drive local adaptation, though the molecular genetic basis remains elusive. Here, we describe a study in which we used genome-wide association mapping, genetic complementation, and gene expression studies to identify cis-regulatory expression level polymorphisms at the *AtHKT1;1* locus, encoding a known sodium (Na^+^) transporter, as being a major factor controlling natural variation in leaf Na^+^ accumulation capacity across the global *A. thaliana* population. A weak allele of *AtHKT1;1* that drives elevated leaf Na^+^ in this population has been previously linked to elevated salinity tolerance. Inspection of the geographical distribution of this allele revealed its significant enrichment in populations associated with the coast and saline soils in Europe. The fixation of this weak *AtHKT1;1* allele in these populations is genetic evidence supporting local adaptation to these potentially saline impacted environments.

## Author Summary

The unusual geographical distribution of certain animal and plant species has provided puzzling questions to the scientific community regarding the interrelationship of evolutionary and geographic histories for generations. With DNA sequencing, such puzzles have now extended to the geographical distribution of genetic variation within a species. Here, we explain one such puzzle in the European population of *Arabidopsis thaliana*, where we find that a version of a gene encoding for a sodium-transporter with reduced function is almost uniquely found in populations of this plant growing close to the coast or on known saline soils. This version of the gene has previously been linked with elevated salinity tolerance, and its unusual distribution in populations of plants growing in coastal regions and on saline soils suggests that it is playing a role in adapting these plants to the elevated salinity of their local environment.

## Introduction

Uncovering the genetic polymorphisms that underlie adaptation to environmental gradients is a critical goal in evolutionary biology, and will lead to a better understanding of both the types of genetic changes and the gene functions involved. Such understanding will not only provide insight into how organisms may respond to future global climate change, but will also provide tools for the development of agricultural systems and ecological services that are more resilient to such changes.

Patterns of phenotypic diversity across environmental gradients can be indicative of adaptive responses to selection, and evaluation of these patterns has the potential to lead to the identification of the genetic polymorphisms underlying these adaptive responses. Numerous studies in animals and plants have identified phenotypic clines in various life history traits, but only a few have determined the genetic changes driving such traits. In *Arabidopsis thaliana*, plasticity in seasonally regulated flowering appears to be modulated by a network of gene interactions responsive to both vernalization and photoperiod signals [1]. Adaptive clines in resistance to oxidative stress and chilling [2], and wing size [3] in *Drosophila melanogaster* are modulated by the *Insulin-like Receptor* (*InR*) and *Drosophila cold acclimation* (*Dca*) genes, respectively. While adaptation to high altitude in *Peromyscus maniculatus* (Deer mice) is associated with enhanced pulmonary O_2_ loading driven by alterations in α-globin and β-globin genes [4]. These genetic changes are all associated with adaptation to variation in environmental factors that vary with latitude or altitude. Such systematic variation has greatly facilitated the discovery of these loci and their adaptive significance. Clines in various life history traits have also been identified in plants growing on serpentine [5], saline [6], [7], and mine impacted soils [8]. Progress has been made in outlining the genetic architecture of these adaptive traits [5], [8]–[10], though a molecular genetic understanding is still needed.

*A. thaliana* is broadly distributed in its native Europe and central Asia, where it experiences a wide range of altitudinal, climatic, and edaphic conditions, leading to a range of selective pressures [11]. Whether the wide variety of natural phenotypic and genetic variation observed in *A. thaliana* [12] contributes to its local adaptation is an important unresolved question that is currently attracting a significant amount of attention [13].

Because of its relevance to crop production, salinity tolerance in plants has been studied intensively [14], and natural plant populations adapted to such conditions have provided an excellent system for studying the evolutionary mechanisms of adaptation and speciation in coastal [6], [10] and salt marsh [7], [9], [15]–[18] environments. The primary effects of excess Na^+^ on plants are water deficit resulting from a water potential gradient between the soil solution and plant cells, and cytotoxicity due of intracellular Na^+^ accumulation [14]. To overcome these effects plants must both accumulate solutes for osmotic regulation, and detoxify intracellular Na^+^ either by limiting its accumulation, or by compartmentalizing Na^+^ into the vacuole. In addition, Na^+^ compartmentalization facilitates vacuolar osmotic adjustment that is necessary to compensate for the osmotic effects of salinity by maintaining turgor pressure for cell expansion and growth. Plants therefore need to strike a balance between the accumulation of Na^+^ to maintain turgor, and the need to avoid Na^+^ chemical toxicity, and this balance will depend in part on soil salinity levels. Given the critical role Na^+^ accumulation plays in salinity tolerance, we used this life history trait to probe the global *A. thaliana* population for signals of adaptive selection for growth in saline impacted environments.

## Results/Discussion

We grew 349 accessions of *A. thaliana* in a controlled common garden in non-saline soil, and analyzed leaf Na^+^ accumulation. We observed a wide range of leaf Na^+^ accumulation across the accessions (330–4,848 mg kg^−1^ dry weight). If this natural variation in leaf Na^+^ accumulation capacity is related to adaptation to growth in saline soils we would expect to find evidence of an adaptive cline, or a gradient of leaf Na^+^ accumulation that correlates with the geographical distribution of variation in soil salinity. Salinity impacted soils are expected to occur in coastal regions due to air born deposition of sea spray which can occur many tens of km inland [19]–[22], but can also occur in areas distant from the coast through high Na^+^ in the soil or ground water. Elevated soil salinity can also be caused by inappropriate irrigation practices such as irrigation with saline water or poor drainage.

**Figure 1:**
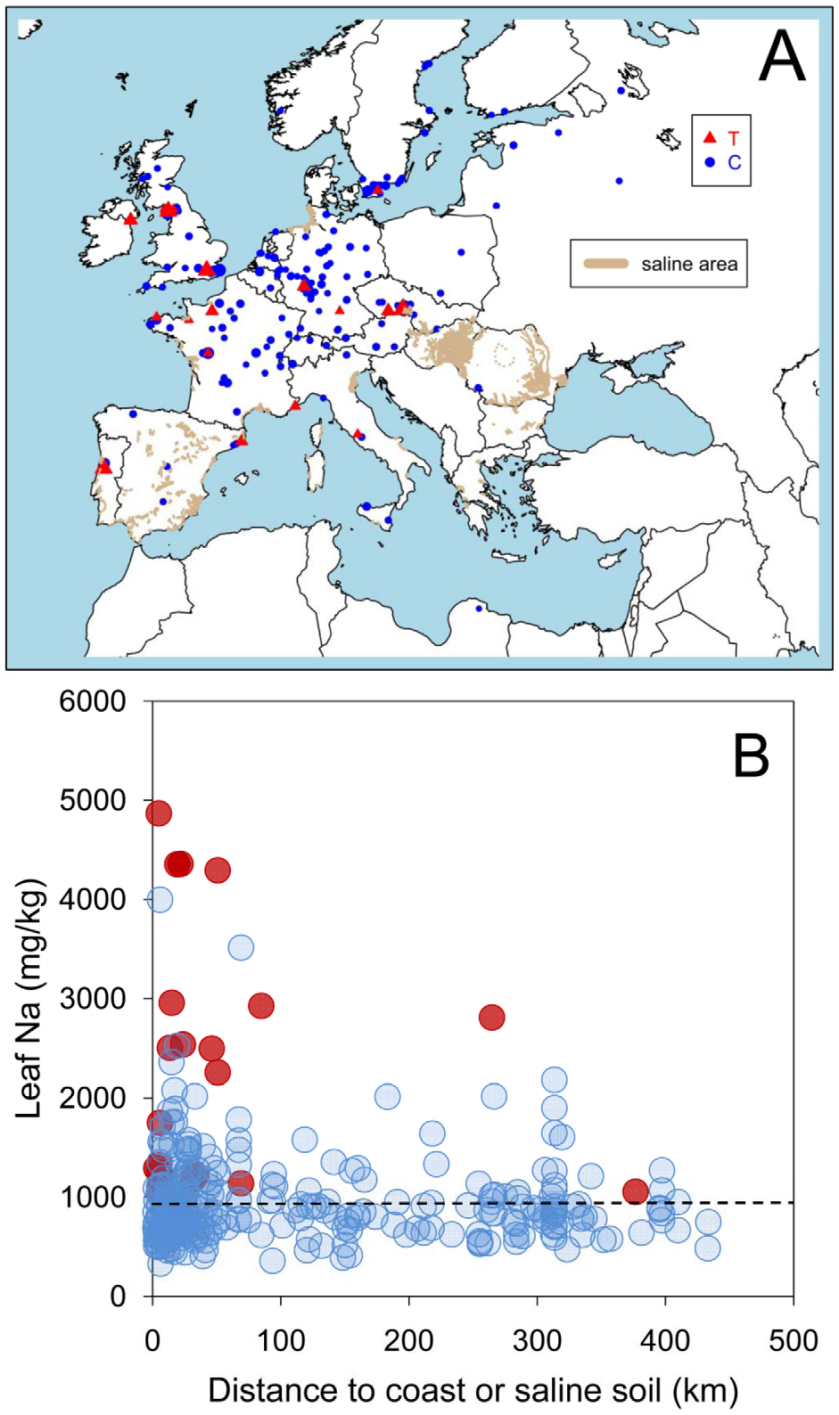
A coastal/saline soil cline in leaf Na^+^ accumulation in *A. thaliana* driven by natural variation at the *AtHKT1;1* locus. A. Map showing the geographical position for the collection site of 300 accessions of *A. thaliana*. The genotype at *Chr4:6392276* of each accession is represented by the type of symbol (red triangle *Chr4:6392276* = T, blue circle *Chr4:6392276* = C). Leaf Na^+^ accumulation of each accession, measured in a common garden experiment, is represented by the size of the symbols. Known areas of saline soils were obtained from Tóth et al., [23] and are represented here in green. B. Leaf Na^+^ accumulation in 300 accessions of *A. thaliana*, measured in a common garden experiment, and its relationship with the distance to the collection site of each accession to the coast or known saline soils (whichever is the smallest). Red symbols genotype at *Chr4:6392276* = T, blue symbols genotype at *Chr4:6392276* = C. Dashed black line represents the average leaf Na^+^ for the Col-0 accession.

To test for the existence of an adaptive cline in leaf Na^+^ accumulation capacity and soil salinity we related leaf Na^+^ accumulation capacity to the distance of the collection site for each accession to the coast, or to the nearest known saline soil, whichever is the shortest. We focused on European accessions since a good soil salinity map exists for this region [23], which left 300 accessions. Regressing the distance to the coast, or nearest known saline soil, on leaf Na^+^ for all 300 accessions revealed a significant relationship (p-value<2e-12), establishing that accessions with elevated leaf Na^+^ are more likely to grow in potentially saline impacted soils (Figure 1A and 1B).

**Figure 2:**
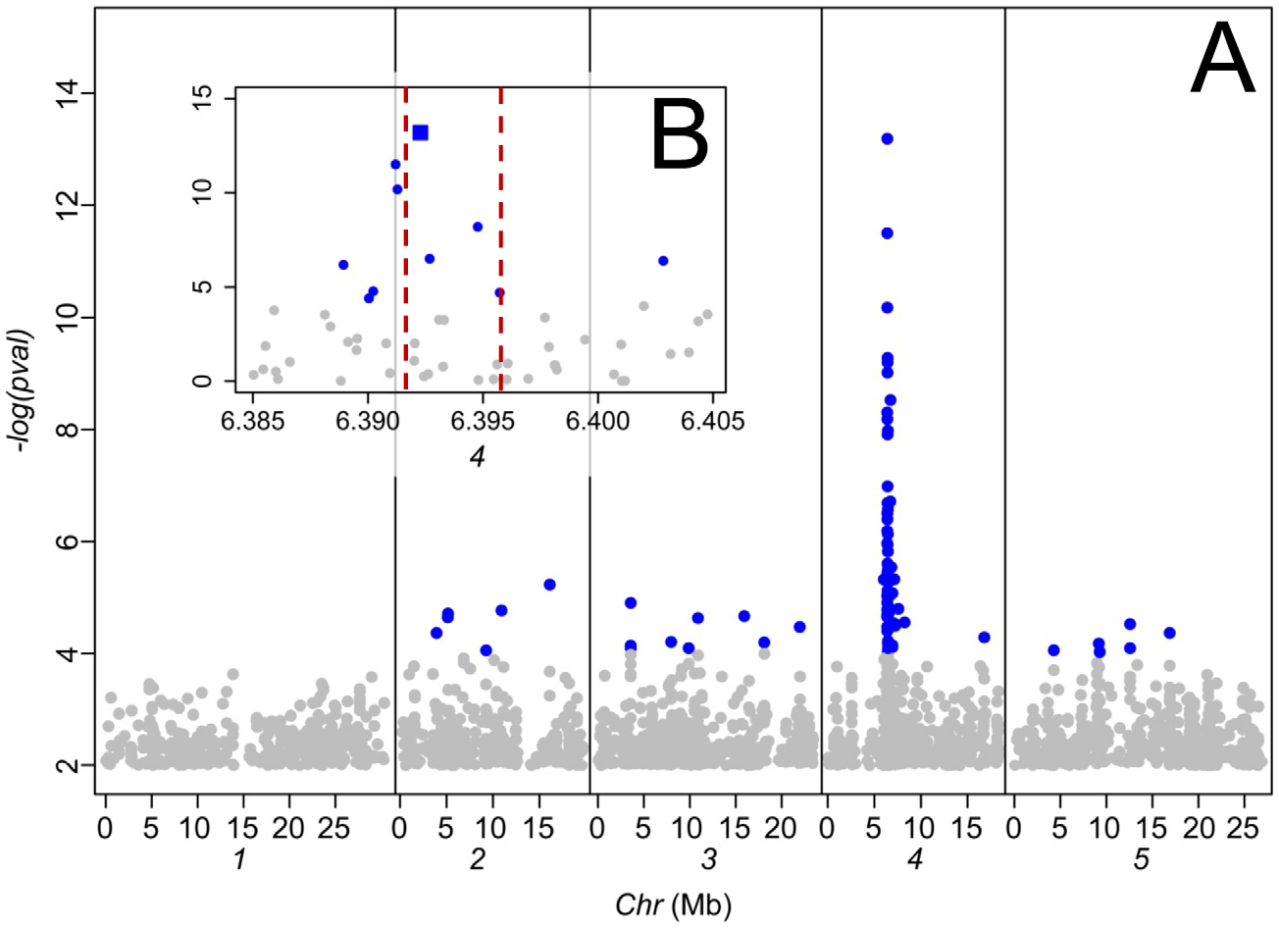
Genome-wide association analysis of leaf Na^+^ accumulation in 349 *A. thaliana* accessions grown in a common garden. A. Genome-wide p-values from a mixed model analysis implemented in EMMA [24]. Associations with a p-value>0.001 are shaded in grey. B. Magnification of the genomic region surrounding *AtHKT1;1*, the position and extent of which is indicated by vertical lines (locus At4g10310 Chr4:6391984–6395877). The SNP most significantly associated with leaf Na^+^ accumulation (*Chr4:6392276*) is represented by a square symbol, all other SNPs represented by circles. Mb, megabase.

To investigate the genetic architecture underlying this cline in leaf Na^+^ accumulation capacity we performed a genome-wide association (GWA) study (previously described for a smaller data set [24]) to identify regions of the genome at which genetic variation is associated with leaf Na^+^ accumulation capacity. The 337 *A. thaliana* accessions used in our GWA study, which are a subset of the 349 accessions phenotyped for leaf Na^+^, were genotyped using the Affymetrix SNP-tilling array Atsnptile1a which can interrogate 248,584 SNPs. To assess evidence of association between SNPs and leaf Na^+^ accumulation we used a mixed-model approach [25] to correct for population structure, as previously described [24]. In the current analysis we identified a single strong peak of SNPs associated with leaf Na^+^, with the peak centered on *AtHKT1;1* (Figure 2), a gene known to encode a Na^+^-transporter [26]. Accessions with a thymine (T) at the SNP most significantly associated with leaf Na^+^ at position 6392276 bp on chromosome 4 (*Chr4:6392276*) have significantly higher leaf Na^+^ than accessions with a cytosine (C) at this same position (2,325 vs. 955 mg Na^+^ kg^−1^ dry weight, p-value<2e-16). This SNP explains 32% (without accounting for population structure) of the total variation in leaf Na^+^ accumulation observed.

**Figure 3:**
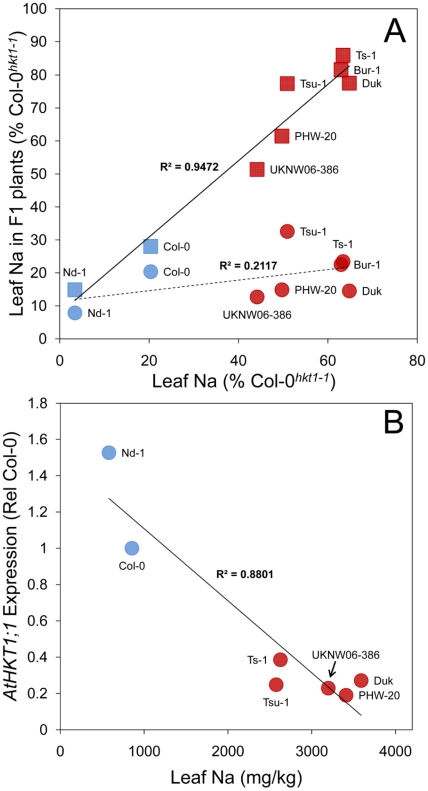
*AtHKT1;1* allele strength in various *A. thaliana* accessions assessed by genetic complementation and expression level. A. Leaf Na^+^ accumulation of F1 plants generated from crosses of various accessions to either Col-0*^hkt1-1^* (squares) or Col-0^HKT1^ (circles) compared to leaf Na^+^ accumulation of the non-Col parent of the cross, and represented as a percentage of leaf Na^+^ accumulation of Col-0*^hkt1-1^*. Solid line represents linear regression of data represented by square symbols, dashed line represents linear regression of data represented by circular symbols. B. Root expression level of *AtHKT1;1* in various accessions as determined by qRT-PCR, represented as relative to *AtHKT1;1* expression in Col-0, and compared to leaf Na^+^ accumulation. Red symbols genotype at *Chr4:6392276* = T, blue symbols genotype at *Chr4:6392276* = C.

Previously, in independent test crosses between the high leaf Na^+^ accessions Ts-1 and Tsu-1 (both containing a T at *Chr4:6392276*) and the low leaf Na^+^ accession Col-0 (containing a C at *Chr4:6392276*) QTLs for leaf Na^+^ centered on *AtHKT1;1* were identified in both F2 populations [27]. Such genetic evidence provides independent support that the peak of SNPs associated with leaf Na^+^ observed in our GWA analysis, centered at *AtHKT1;1* (Figure 2), represents a true positive association and not a false positive driven by the high degree of population structure known to exist in *A. thaliana* [24]. Reduced expression of *AtHKT1;1* in Ts-1 and Tsu-1 was concluded to drive the elevated leaf Na^+^ observed in these two accessions [27]. Here, we expand on this observation by establishing the strength of the *AtHKT1;1* alleles in four further high Na^+^ accumulating accessions (Bur-1, Duk, PHW-20 and UKNW06-386) that all contain a T at *Chr4:6392276*, along with a low leaf Na^+^ accession (Nd-1) with a C at *Chr4:6392276*. By examining the leaf Na^+^ accumulation in F1 plants from crosses of each of these accessions to Col-0*^hkt1-1^* and Col-0^HKT1^, we were able to establish a significant correlation between leaf Na^+^ accumulation and the strength of the *AtHKT1;1* alleles (Figure 3A). These crosses confirmed that all accessions tested with elevated leaf Na^+^, and that contain a T at *Chr4:6392276*, have hypofunctional alleles of *AtHKT1;1* relative to the Col-0 allele. Furthermore, analysis of the expression of *AtHKT1;1* in the same set of accessions revealed that allelic variation in *AtHKT1;1* strength is modulated at the level of gene expression (Figure 3B), consistent with what was previously observed for Ts-1 and Tsu-1 [27]. Though the SNP most significantly associated with leaf Na^+^ (*Chr4:6392276*) is unlikely to be causal for these *AtHKT1;1* expression level polymorphisms, this SNP can be used as a linked genetic marker to determine the type of *AtHKT1;1* allele present, with a T at this SNP being associated with weak *AtHKT1;1* alleles.

Using the SNP at *Chr4:6392276* as a genetic marker for the type of *AtHKT1;1* allele (strong or weak) allowed us to test the hypothesis that the leaf Na^+^ soil salinity cline we observe in European populations of *A. thaliana* (Figure 1A and 1B) is associated with weak alleles of *AtHKT1;1*. By comparing the means of distances to the coast, or known saline soil, for the collection site of all 300 accessions with and without a T at *Chr4:6392276*, we determined that a significant association (parametric test p-value = 0.0001; non-parametric Wilcoxon rank-sum test p-value = 0.0062) exists between *A. thaliana* growing on potentially saline impacted soils and the presence of a weak allele of *AtHKT1;1* (Figure 1A and 1B). Such a strong correlation between the presence of allelic variation at *AtHKT1;1* known to drive elevated leaf Na^+^, and the observed cline in leaf Na^+^ and saline soils, is evidence for the involvement of *AtHKT1;1* in determining this geographical distribution. Furthermore, using 13 SNPs within a 20kb region centered on *HKT1;1* to define the *HKT1;1* haplotype, we identify 7 haplotypes (6 if you combine haplotypes with only 1 SNP different) in accessions with high leaf Na^+^ (>2,500 ppm), suggesting that weak alleles of *HKT1;1* have arisen independently multiple times.

However, to be credible it is also important to provide evidence that selection for growth on saline soils could be acting on the phenotype driven by allelic variation at *AtHKT1;1*; in this case elevated leaf Na^+^. Such evidence is provided by the previous observation that the weak allele of *AtHKT1;1* in the coastal Tsu-1 *A. thaliana* accession not only causes elevated leaf Na^+^ but is also genetically linked to the elevated salinity tolerance of this accession [27].

In *A. thaliana* AtHKT1;1 functions to unload Na^+^ from xylem vessels in the root, controlling translocation and accumulation of Na^+^ in the shoots [26], [28]. Therefore, modulation of its function would allow the balancing of Na^+^ accumulation in the shoot with soil salinity. We note here that the *hkt1-1* null mutation in the Col-0 background causes plants to exhibit dramatic leaf Na^+^ hyperaccumulation and increased NaCl sensitivity [29], [30]. We interpret this to mean that expression of *AtHKT1;1* in the *hkt1-1* null mutant is reduced to such an extent that leaf Na^+^ accumulation saturates the capacity for cellular detoxification of Na^+^ by vacuolar compartmentalization. We propose that the naturally occurring weak alleles of *AtHKT1;1*, that we show are associated with populations growing in potentially saline impacted environments, allow sufficient Na^+^ to accumulate in leaves for osmotic adjustment, conferring elevated Na^+^ tolerance. However, these weak, but not complete loss-of-function *AtHKT1;1* alleles, do not saturate the mechanism whereby the accessions avoid Na^+^ cytotoxicity. The basis of this Na^+^ detoxification mechanism remains to be determined, though an active leaf vacuolar Na^+^ compartmentalization mechanism driven by AtNHX1 is one likely candidate.

In conclusion, here we provide evidence supporting the involvement of specific cis-regulatory polymorphisms at *AtHKT1;1* in the potentially adaptive cline in leaf Na^+^ accumulation capacity we observe in *A. thaliana* populations to saline impacted environments. We have identified a strong association between the *AtHKT1;1* allele frequency in *A. thaliana* populations and their growth on potentially saline impacted soils (Figure 1A and 1B). Further, we have confirmed by GWA mapping, experimental complementation crosses, and gene expression studies, that this allelic variation directly causes changes in the clinally varying leaf Na^+^ accumulation phenotype via cis-regulatory polymorphisms (Figure 2 and Figure 3). And, finally, we have previously established that the weak *AtHKT1;1* alleles we show to be associated with potentially saline soils, are also linked to elevated salinity tolerance [27], providing a plausible mechanistic link between selection for growth on saline soils and variation in *AtHKT1;1* allele frequency. Such discoveries provide tantalizing evidence that points to selection acting at *AtHKT1;1* in natural populations of *A. thaliana* in adaptation to growth in saline environments.

## Materials and Methods

### Plant Material and Plant Growth Conditions

Plants were grown in a controlled environment with 10 h light/14 h dark (90 µmol m^−2^s^−1^ photosynthetically active light) and 19 to 22°C, as previously described [31]. Briefly, seeds were sown onto moist soil (Promix; Premier Horticulture) in 10.5^″^×21^″^ 20 row trays with various elements added to the soil at subtoxic concentrations (As, Cd, Co, Li, Ni, Rb, and Se [31]) and the tray placed at 4°C for 3 days to stratify the seeds and help synchronize germination. Each tray contained 108 plants, six plants each from 18 accessions, with three plants of each accession planted in two different parts of the tray. Each tray contained four common accessions (Col-0, Cvi-0, Fab-2 and Ts-1) used as controls, and 14 test accessions. Trays were bottom-watered twice per week with 0.25-strength Hoagland solution in which Fe was replaced with 10 µM Fe-HBED[N,N′-di(2-hydroxybenzyl)ethylenediamine-N,N′-diacetic acid monohydrochloride hydrate; Strem Chemicals, Inc.). After 5 weeks plants were non-destructively sampled by removing one or two leaves and the elemental composition of the tissue analyzed by Inductively Couple Plasma Mass Spectroscopy (ICP-MS). The plant material was rinsed with 18 MΩ water and placed into Pyrex digestion tubes. For complementation experiments plants were crossed to Col-0 or Col-0*^hkt1-1^* and approximately 12 F1 plants were grown in the conditions described above.

### Mapping Population

A set of 360 *A. thaliana* accessions were selected from 5,810 worldwide accessions to minimizing redundancy and close family relatedness, based on the genotypes at 149 SNPs developed in a previous study [32]. Figure S1 and Table S1 show the genetic variation in the core set of 360 accessions vs. a random set of 360 accessions chosen from the genotyped 5,810 accessions. From the selected core set of 360 accessions a subset of 349 were phenotyped using ICP-MS, and of these 337 were genotyped using the Affymetrix SNP-tilling array Atsnptile1 which contains probe sets for 248,584 SNPs. Details of the SNP-tilling array and methods for array hybridization and SNP-calling are the same as previously described [24]. In brief, approximately 250 ng of genomic DNA was labeled using the BioPrime DNA labeling system (Invitrogen) and 16 µg of the labelled product hybridized to each array. SNPs were called using the Oligo package after slight modifications. Quality control (QC) of the genotypes, and imputation of the missing SNPs were performed following the procedure previously described [24], except that a 15% mismatch rate was used to filter out low quality arrays. After QC and imputation, the 337 accessions had genotypes for at least 213,497 SNPs. The core set of 360 accessions selected are all available from the Arabidopsis Biological Resource Center (http://abrc.osu.edu/), and the SNP genotypes for the 337 accessions used for the GWA study are available from http://borevitzlab.uchicago.edu/resources/genetic/hapmap/BaxterCore/.

### Tissue Elemental Analysis

Samples were analyzed as described by Lahner *et al.* [31]. Tissue samples were dried at 92°C for 20 h in Pyrex tubes (16×100 mm) to yield approximately 2–4 mg of tissue for elemental analysis. After cooling, seven of the 108 samples from each sample set were weighed. All samples were digested with 0.7 ml of concentrated nitric acid (OmniTrace; VWR Scientific Products), and diluted to 6.0 ml with 18 MΩ water. Elemental analysis was performed with an ICP-MS (Elan DRCe; PerkinElmer) for Li, B, Na, Mg, P, S, K, Ca, Mn, Fe, Co, Ni, Cu, Zn, As, Se, Rb, Mo, and Cd. A liquid reference material composed of pooled samples of *A. thaliana* leaves was run every 9*^th^* sample to correct for ICP-MS run to run variation and within-run drift. All samples were normalized to the calculated weights, as determined with an iterative algorithm using the best-measured elements, the weights of the seven weighed samples, and the solution concentrations, implemented in the Purdue Ionomics Information Management System (PiiMS) [33] (for a full description see www.ionomicshub.org). Data for all elements is available for viewing and download at www.ionomicshub.org in trays 1478–1504.

### Quantification of *AtHKT1;1* mRNA

To quantify the levels of *AtHKT1;1* mRNA in roots of the various accessions studied, we used a protocol similar to that of Rus *et al.* [27]. Roots from plants grown under identical conditions to those used for ICP-MS analysis were separated from the shoots and rinsed thoroughly with deionized water to remove any soil contamination. The samples were frozen in liquid nitrogen and stored at −80°C until extraction. Total RNA was extracted, and DNase digestion was performed during the extraction, using the Invitrogen PureLink RNA Mini Kit. Two micrograms of total RNA were used as a template to synthesize first-strand cDNA with random hexamers, using SuperScript II Reverse Transcriptase (Invitrogen Life Technologies). Quantitative real-time PCR (qRT-PCR) was performed with first strand cDNA as a template on four technical replicates from three independent biological samples for each accession, using a sequence detector system (StepOne Plus, Applied Biosystems). For normalization across samples within a qRT-PCR run the expression of the *Actin 1* gene (At2g37620) was used with the following primers: CPRD66, 5′-TGG AAC TGG AAT GGT TAA GGC TG-3′ and CPRD67, 5′-TCT CCA GAG TCG AGC ACA ATA C-3′. For quantification of *AtHKT1;1* the following primers were used: HKT-RTF, 5′-TGG GAT CTT ATA ATT CGG ACA GTT C-3′ and HKT-RTR, 5′-GAT AAG ACC CTC GCG ATA ATC AGT-3′. The fold induction relative to *AtHKT1;1* expression in Col-0 roots was calculated following the method of Livak and Schmittgen [34]. CT values were determined based on efficiency of amplification. The mean CT values were normalized against the corresponding *Actin 1* gene and ΔCT values calculated as CT*_AtHKT1;1_*–CT*_Actin 1_*. The expression of *AtHKT1;1* was calculated using the 2^∧(ΔCT)^ method [34]. To normalize between samples analyzed in separate qRT-PCR runs, we divided the ΔCT for each line by the ΔCT of Col-0 roots in that run.

### Data Analysis

ICP-MS measurements below zero and extreme outliers (those values that were greater than the 90*^th^* percentile +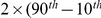 percentile) within each tray were removed. To account for variation in the growth environment, the four control accessions included in each tray were used to create a tray specific normalization factor. Briefly, for each element, each control accession in a given tray was compared to the overall average for that accession across all trays to obtain an element×line×tray specific normalization factor. The four element×line×tray factors in a give tray were then averaged to create a tray×element normalization factor for the tray. Every value for the element in the tray was then multiplied by the normalization factor. See Figure S2 for data of control accessions before and after the normalization. The mean of each accession was then used for all subsequent analysis. Normalized Na^+^ values and their frequency distribution can be found in Dataset S1 and Figure S3.

Genotype calls for all 349 accessions were obtained using the methods previously described [24]. GWA analysis was done with correction for confounding using a mixed-model that uses a genetic random effect with a fixed covariance structure to account for population structure [25] implemented in the program EMMA [24]. The contribution of the best performing SNP (C or T at *Chr4:6392276 = isT*) was checked using un-normalized Na^+^ data and the linear model:
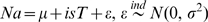
(1)using the *lm* and *anova* functions from R v2.9.1. The control accessions were excluded from this analysis. The output of the statistical model can be found in Text S1. Although the samples were nested in trays, Figure S4 indicates that the best performing SNP is essentially evenly distributed across all trays.

The geographical location of each accession was obtained from TAIR (www.arabidopsis.org). When processing the original data, we found an inconsistency for one of the high-Na accessions, CS28373 (also known as Jm-1). The listed latitude and longitude (49, 15) of the accession do not match the location name “Jamolice” from where this accession was collected. The town Jamolice is located at 49.0721283 latitude and 16.2532139 longitude (http://www.gpsvisualizer.com/geocode). In the interests of consistency, we used the original coordinates, although altering the location did not materially change the analysis. The distance to the coast or saline/sodic areas was calculated by obtaining the longitudes and latitudes of the shoreline/coast from the National Oceanic and Atmospheric Administration's National Geophysical Data Center (NOAAs NGDC http://www.ngdc.noaa.gov/ngdc.html) and the saline and sodic soils data from the European Soils database [23]. The *pointDistance* function in R 2.10.0 and the package *raster* were used to calculate the Great-circle distance to the shoreline or saline/sodic areas. We created a variable (*toSeaSal*) representing the shortest distance from the target accession to the shoreline/coast or saline/sodic area. The accession coordinates, distance to sea, distance to saline environment and SNP genotype at *Chr4:6392276* can all be found in Dataset S1.

The method used to collect accessions and assemble the population might introduce unintended confounding effects that violate the assumption of independent locations used by our models. To determine whether the locations of the accessions were spatially dependent we performed a Mantel test [35] on the distances from the 300 accessions to the coast or known saline/sodic areas. The simulated p-values of 50 permutations tests with 999 repeatedly simulated samples are 0.996, indicating that an assumption of independency for the response variable *toSeaSal* is acceptable.

To test for associations between leaf Na^+^ (*Na*), genotype at the highest scoring SNP (C or T at *Chr4:6392276* = *isT*), and the distance to the nearest coast or saline/sodic area (*toSeaSa*l), we used the package *lm* in R 2.10.0 to fit linear models, with the weights determined by the following approach. First, to quantify the strength of the relationship between *toSeaSal* and the leaf sodium *Na*, we fit the data into a linear model and regressed *toSeaSal* on *Na*.
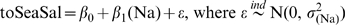
(2)Second, we applied a regression approach to single-factor analysis [36] between *toSeaSal* and *isT* and tested if the average distance to coast or saline/sodic areas of samples having the high Na T allele is significantly different from the average of samples having the C allele.
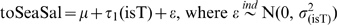
(3)Finally, we regressed *toSeaSal* on the interaction between *Na* and *isT* to inspect how the two predictors jointly affect the distance to sea or saline/sodic.
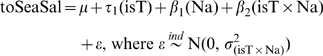
(4)To perform the significance tests on the linear coefficients, *Na* should be centered at the mean [36]. The extent of variation of distances to saline environments changes with both leaf Na^+^ concentrations and genotypes (Figure S5). Therefore, all three models account for this heterogeneity of variation, and parameters of the models are fitted using weighted least squares. The variances of the error terms in equation 2, 3, and 4 are not constant, and are related to the predictors according to the diagnosis on the model residuals. The models were fit using iterative weighted least squares [36]. In addition to the parametric test (model 3), we performed a non-parametric test (Wilcoxon rank-sum test or Wilcoxon-Mann-Whitney test [37]) using the *wilcox.test* function in R package *stats*, to assess whether *toSeaSal* is higher in the lines with the T allele than those with the C allele at *Chr4:6392276*. The p-value of the Wilcoxon rank-sum test is 0.006224 indicating that both the parametric and non-parametric approaches reach the same conclusion. The statistical output of all models can be found in Text S1.

## Supporting Information

Dataset S1 Data set containing accession name, ID, country and origin, lat and long of collection site, leaf Na, SNP at C4_6392276, toSeaSal, toSea, and toSal.
(0.09 MB XLS)

Figure S1 Pairwise genetic distance for the core 360.
(0.85 MB PDF)

Figure S2 Variation in control lines across trays. The average Na^+^ levels of the four control lines (Col-0, Cvi-0, Fab-2 and Ts-1) as well as the average of all other lines in each tray is plotted before (A) and after (B) normalization.
(0.25 MB PPTX)

Figure S3 The distribution of average leaf Na^+^ values across all accessions used for association mapping. The x-axis displays the parts per million (ppm) Na^+^ values before normalization. The barbell symbols indicate the range of the Na values for the four control accessions across the 26 trays of the experiment.
(0.10 MB PPTX)

Figure S4 Distribution of Chr4:6392276 across experiments. The lines containing the high Na^+^ allele at the SNP most significantly correlated with Na (Chr4:6392276) are approximately evenly distributed among the 26 trays of the experiment. Bars indicate the number of accessions having either the T allele (blue) or the C allele (red) at the SNP most significantly correlated with Na^+^, Chr4:6392276.
(0.16 MB PPTX)

Figure S5 Scatter plot of distance to sea or saline/sodic areas (toSeaSal) versus leaf Na. The red colored T corresponds to the samples having a T at Chr4:6392276. The blue colored C represents samples having a C at Chr4:6392276. The black line is a lowess curve fitting all the 300 samples. The red line is a lowess curve fitting the 21 samples with the T allele. The blue line is a lowess curve fitting the 279 samples with the C allele.
(0.34 MB PPTX)

Table S1 Comparing the genetic variation between Core360 and Random 360 accessions.
(0.04 MB DOC)

Text S1 Output of statistical models.
(0.01 MB DOCX)
