# Supplementary figures and images for: A Coastal Cline in Sodium Accumulation in Arabidopsis thaliana Is Driven by Natural Variation of the Sodium Transporter AtHKT1;1
Source: PLoS Genet. 2010 Nov 11;6(11):e1001193. doi: 10.1371/journal.pgen.1001193 (PMC2978683; doi:10.1371/journal.pgen.1001193)

**Core360set**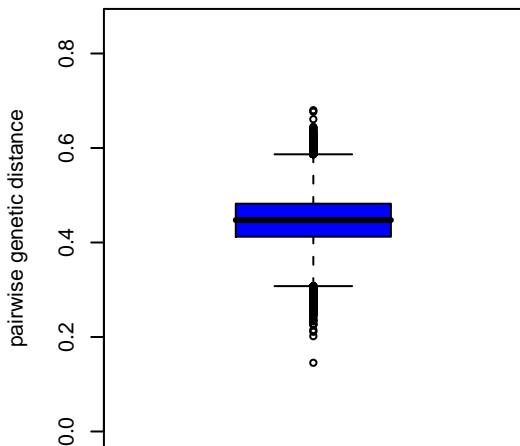**Random360set1**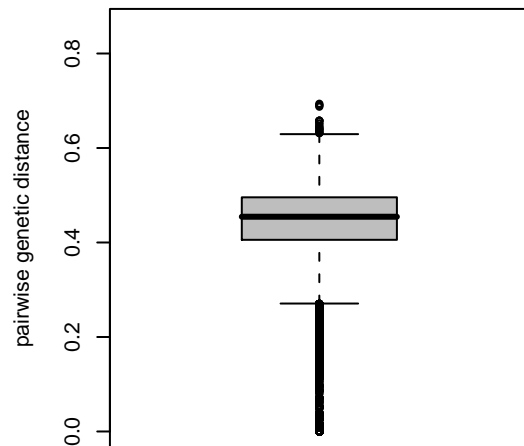**Random360set2**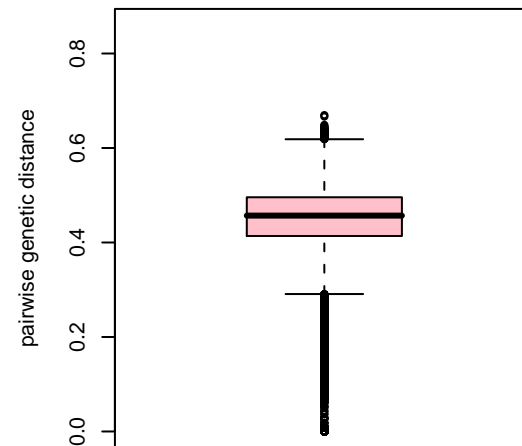**Random360set3**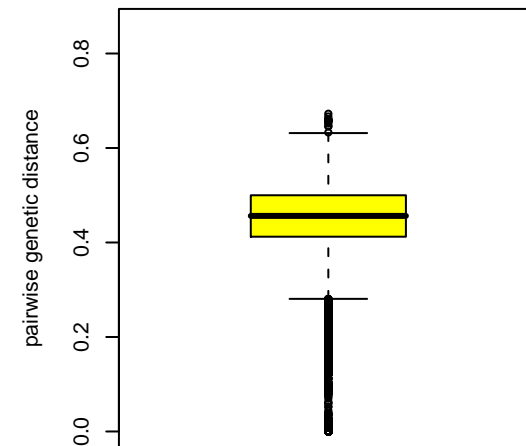**Core360set**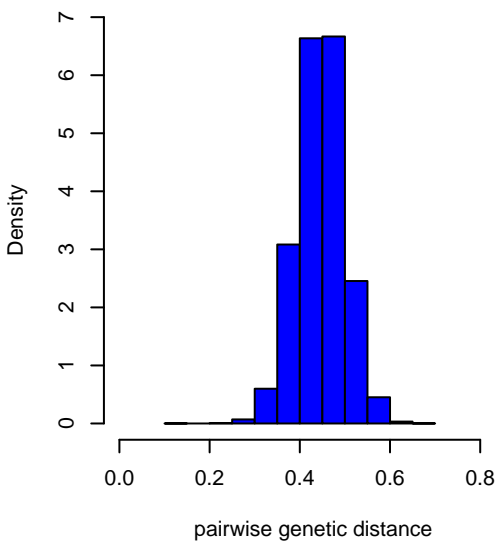**Random360set1**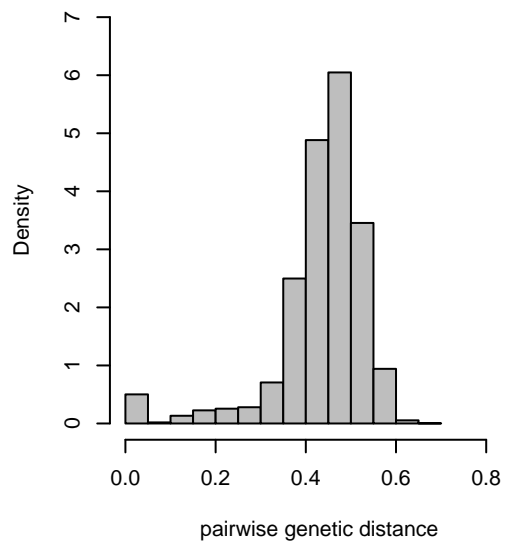**Random360set2**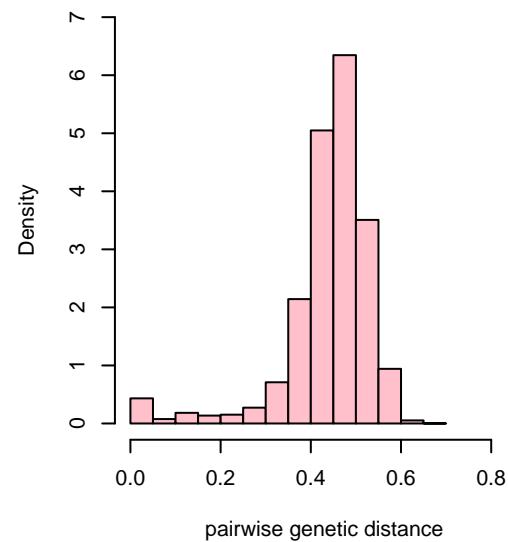**Random360set3**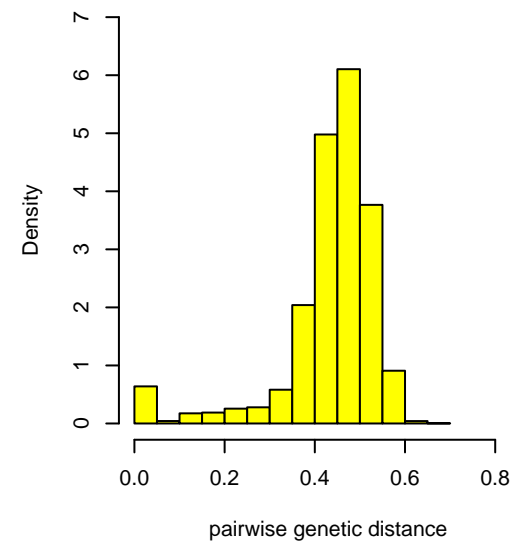

Supplement: Figure S1 — Pairwise genetic distance for the core 360. (0.85 MB PDF) [file pgen.1001193.s002.pdf]

## Slide 1
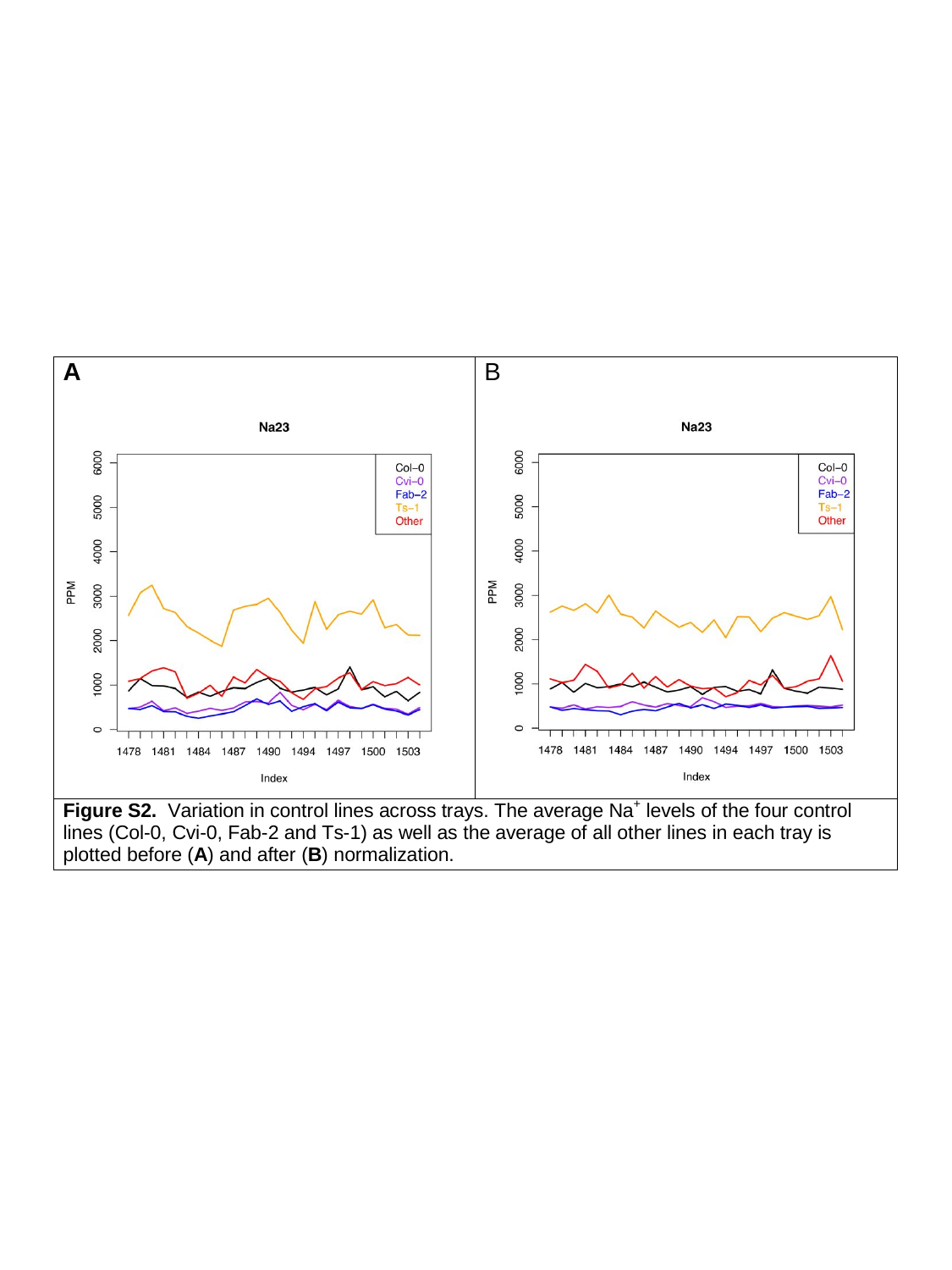

Supplement: Figure S2 — Variation in control lines across trays. The average Na+ levels of the four control lines (Col-0, Cvi-0, Fab-2 and Ts-1) as well as the average of all other lines in each tray is plotted before (A) and after (B) normalization. (0.25 MB PPTX) [file pgen.1001193.s003.pptx]

## Slide 1
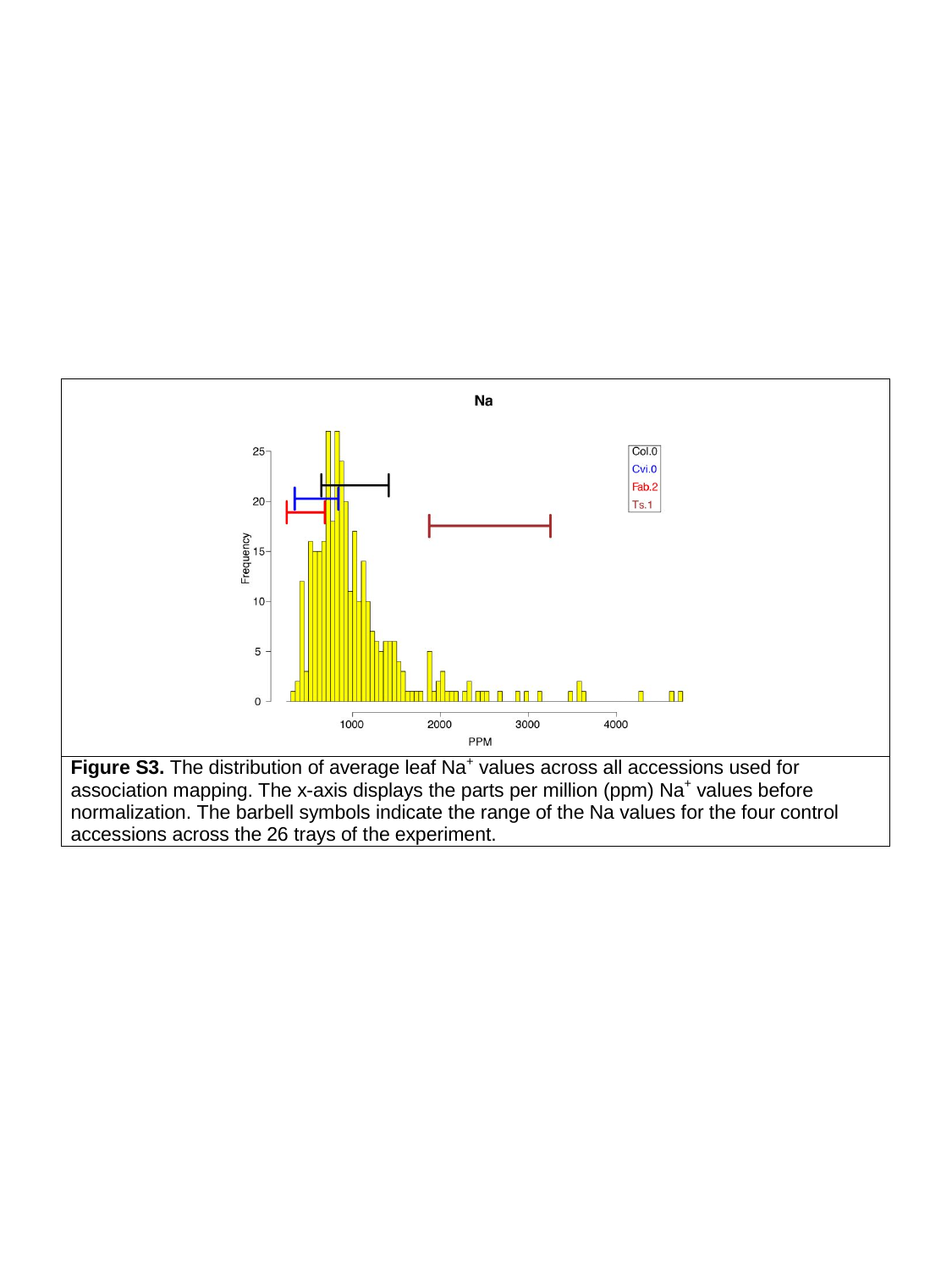

Supplement: Figure S3 — The distribution of average leaf Na+ values across all accessions used for association mapping. The x-axis displays the parts per million (ppm) Na+ values before normalization. The barbell symbols indicate the range of the Na values for the four control accessions across the 26 trays of the experiment. (0.10 MB PPTX) [file pgen.1001193.s004.pptx]

## Slide 1
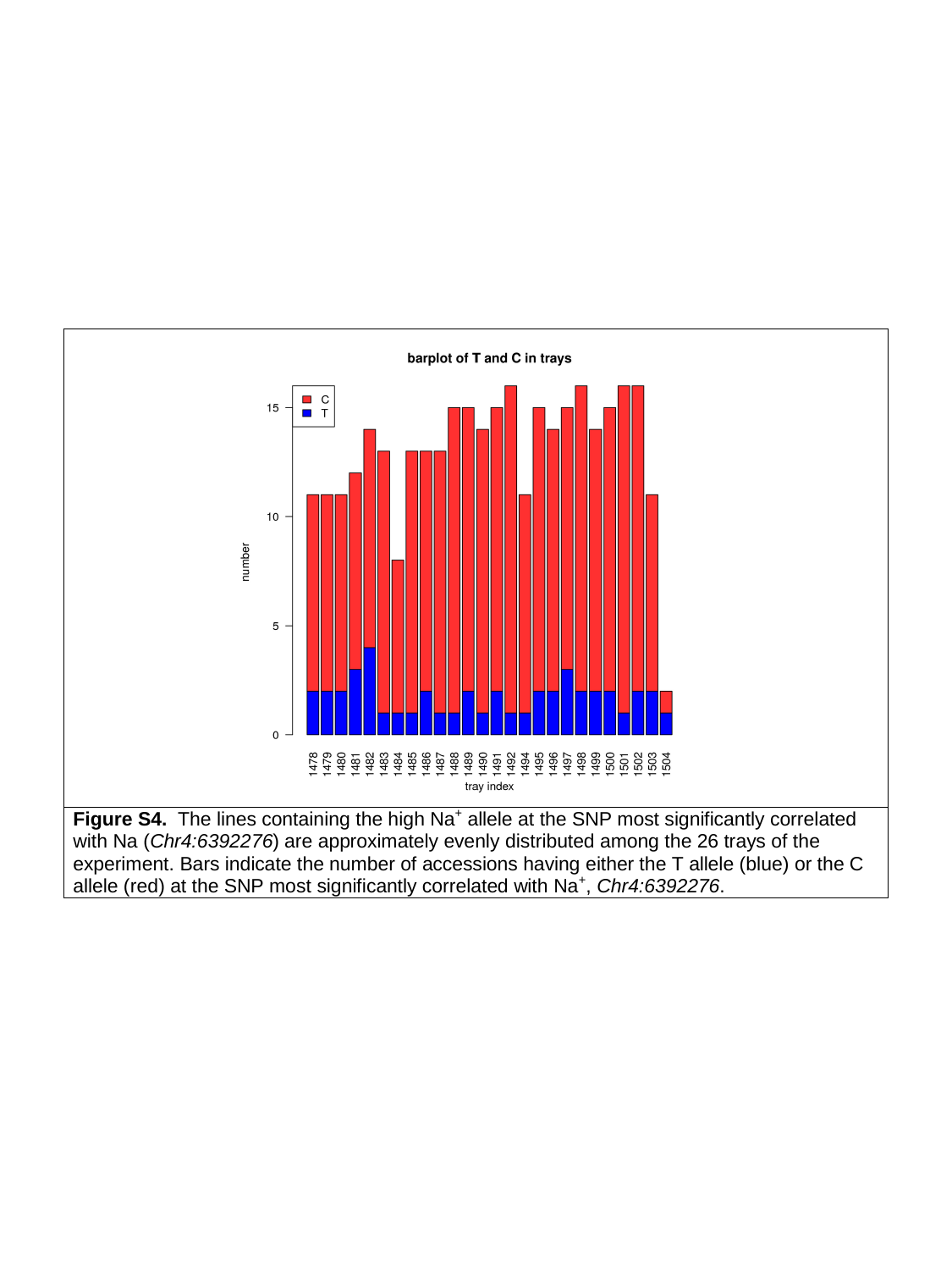

Supplement: Figure S4 — Distribution of Chr4:6392276 across experiments. The lines containing the high Na+ allele at the SNP most significantly correlated with Na (Chr4:6392276) are approximately evenly distributed among the 26 trays of the experiment. Bars indicate the number of accessions having either the T allele (blue) or the C allele (red) at the SNP most significantly correlated with Na+, Chr4:6392276. (0.16 MB PPTX) [file pgen.1001193.s005.pptx]

## Slide 1
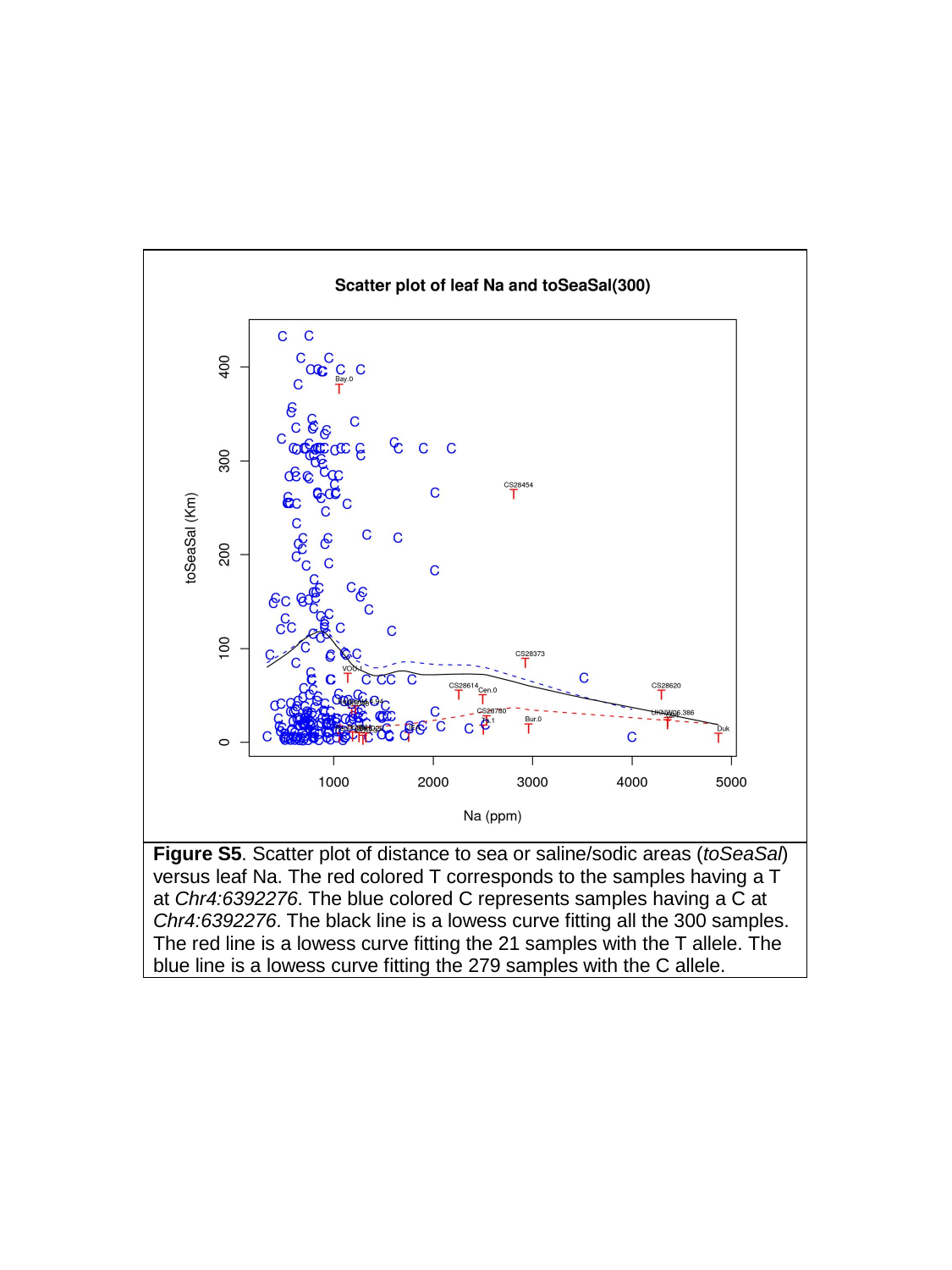

Supplement: Figure S5 — Scatter plot of distance to sea or saline/sodic areas (toSeaSal) versus leaf Na. The red colored T corresponds to the samples having a T at Chr4:6392276. The blue colored C represents samples having a C at Chr4:6392276. The black line is a lowess curve fitting all the 300 samples. The red line is a lowess curve fitting the 21 samples with the T allele. The blue line is a lowess curve fitting the 279 samples with the C allele. (0.34 MB PPTX) [file pgen.1001193.s006.pptx]
