# Supplementary material for: A Coastal Cline in Sodium Accumulation in Arabidopsis thaliana Is Driven by Natural Variation of the Sodium Transporter AtHKT1;1
Source: PLoS Genet. 2010 Nov 11;6(11):e1001193. doi: 10.1371/journal.pgen.1001193 (PMC2978683; doi:10.1371/journal.pgen.1001193)
Supplement: Table S1 — Comparing the genetic variation between Core360 and Random 360 accessions. (0.04 MB DOC) [file pgen.1001193.s007.doc]

**Table S4 Compare the genetic variation between Core360 and Random 360 accessions^*^**

|  | **Core360** | **Random360Set1** | **Random360Set2** | **Random360Set3** |
| --- | --- | --- | --- | --- |
| **total number of pairs** | 64620 | 64620 | 64620 | 64620 |
| **average pairwise genetic distance** | 0.45 | 0.44 | 0.44 | 0.44 |
| **min pairwise genetic distance** | 0.15 | 0 | 0 | 0 |
| **max pairwise genetic distance** | 0.68 | 0.69 | 0.67 | 0.67 |
| **number of haplotypes h0** | 360 | 238 | 228 | 232 |
| **number of haplotypes h0.25** | 350 | 163 | 154 | 166 |
| **number of haplotypes h0.5** | 33 | 19 | 21 | 20 |

^*^117 SNPs x 5810 accessions
