# Supplementary material for: A Coastal Cline in Sodium Accumulation in Arabidopsis thaliana Is Driven by Natural Variation of the Sodium Transporter AtHKT1;1
Source: PLoS Genet. 2010 Nov 11;6(11):e1001193. doi: 10.1371/journal.pgen.1001193 (PMC2978683; doi:10.1371/journal.pgen.1001193)
Supplement: Text S1 — Output of statistical models. (0.01 MB DOCX) [file pgen.1001193.s008.docx]

R-output of model 1:

> snp.mod <- lm(small.na.nocon$Na23 ~ isT, small.na.nocon)

> anova(snp.mod)

Analysis of Variance Table

Response: small.na.nocon$Na23

Df Sum Sq Mean Sq F value Pr(>F)

isT 1 42406266 42406266 157.45 < 2.2e-16 ***

Residuals 334 89959527 269340

---

Signif. codes: 0 *** 0.001 ** 0.01 * 0.05 . 0.1 1

> x <- anova(snp.mod)

> x$Sum[1]/sum(x$Sum)

[1] 0.3203718

R-output of model 2:

Call:

lm(formula = toSeaSal ~ Na, weights = 1/weightsForEachObs)

Residuals:

Min 1Q Median 3Q Max

-1.0586 -0.8975 -0.6537 0.9244 3.1132

Coefficients:

Estimate Std. Error t value Pr(>|t|)

(Intercept) 143.05629 9.86209 14.506 < 2e-16 ***

Na -0.02762 0.00376 -7.345 1.98e-12 ***

---

Signif. codes: 0 *** 0.001 ** 0.01 * 0.05 . 0.1 1

Residual standard error: 1.16 on 298 degrees of freedom

Multiple R-squared: 0.1533, Adjusted R-squared: 0.1504

F-statistic: 53.95 on 1 and 298 DF, p-value: 1.983e-12

R-output of model 3:

Call:

lm(formula = toSeaSal ~ isT, weights = 1/weightsForEachObs)

Residuals:

Min 1Q Median 3Q Max

-1.0550 -0.8947 -0.6634 0.8997 5.8528

Coefficients:

Estimate Std. Error t value Pr(>|t|)

(Intercept) 118.427 7.863 15.062 < 2e-16 ***

isT -64.343 16.351 -3.935 0.000104 ***

---

Signif. codes: 0 *** 0.001 ** 0.01 * 0.05 . 0.1 1

Residual standard error: 1.192 on 298 degrees of freedom

Multiple R-squared: 0.0494, Adjusted R-squared: 0.04621

F-statistic: 15.48 on 1 and 298 DF, p-value: 0.0001036

R-output of model 4:

Call:

lm(formula = toSeaSal ~ centeredNa * isT, weights =

1/weightsForEachObs)

Residuals:

Min 1Q Median 3Q Max

-1.0880 -0.9075 -0.6462 0.9470 4.5270

Coefficients:

Estimate Std. Error t value Pr(>|t|)

(Intercept) 115.85592 7.64346 15.158 <2e-16 ***

centeredNa -0.02763 0.01262 -2.190 0.0293 *

isT -38.91215 24.70295 -1.575 0.1163

centeredNa:isT 0.01176 0.01486 0.791 0.4293

---

Signif. codes: 0 *** 0.001 ** 0.01 * 0.05 . 0.1 1

Residual standard error: 1.183 on 296 degrees of freedom

Multiple R-squared: 0.1495, Adjusted R-squared: 0.1409

F-statistic: 17.34 on 3 and 296 DF, p-value: 2.117e-10
